# Effects of fermentation medium on cigar filler

**DOI:** 10.3389/fbioe.2022.1069796

**Published:** 2022-12-16

**Authors:** Cai Wen, Hu Wanrong, Li Pinhe, Liu Jie, Zhang Qianying, Zhou Quanwei, Luo Cheng, Li Dongliang

**Affiliations:** China Tobacco Sichuan Industrial Co., Ltd., Chengdu, China

**Keywords:** cigar, fermentation medium, aromatic component, quality of tobacco leaf, industrial application of fermentation

## Abstract

The addition of medium during industrial fermentation can improve the quality of cigar tobacco leaves after agricultural fermentation. In this study, the cigar filler tobacco “Brazilian Frogstrips YA14” was used as the test material to determine the contents of main chemical components in cigar tobacco leaves after fermentations with the additions of water (control group) and a medium (test group), and the changes in the community structure and abundances of bacteria on tobacco leaves during the fermentation process were analyzed. The results of the study were as follows: 1) During the fermentation process, the protein content of tobacco leaves fluctuated slightly, basically stabilized at 19%–20%. 2) Under the impact of the medium, the total content of main amino acids in tobacco leaves showed a downward trend, and the difference of which between the control group and the test group was the most obvious on the fourth day of fermentation. 3) The change trend of the content of petroleum ether extract in cigar leaves for the control group was not obvious, and the content of petroleum ether extract in the tobacco leaves for the test group decreased by 12.4% under the impact of the medium. 4) After fermentation, the relative content of saturated fatty acids for the control group and the test group all increased, while the relative content of unsaturated fatty acids all decreased. 5) After the addition of the medium, the diversity of bacteria on tobacco leaves changed significantly, the number of OTUs in tobacco leaves increased, and the bacterial community structure changed. This research indicates that after adding the medium to ferment cigar filler, the changes of bacterial community and dominant bacterial group on cigar tobacco leaves have impacts on the contents of chemical components in tobacco leaves, and the fermentation with the addition of medium has a positive effect on improving the quality of tobacco leaves.

## 1 Introduction

Cigar is a kind of tobacco product, which is made by rolling tobacco leaves and is mainly divided into three parts: filler, binder and wrapper (Li et al., 2012). The cigar tobacco leaves need to be used after being alcoholized (Tang et al., 2009). During the process of alcoholization, the quality of cigar tobacco leaves is improved with the action of microorganisms (Tang et al., 2011). Fermentation is an important process in the production of cigar leaves. Some macromolecular substances such as starch and protein are not completely degraded during fermentation, which has a great negative impact on the combustion and quality of cigar tobacco leaves (Jin, 1988).

Fermentation medium refers to the addition of substances other than water to tobacco leaves, which are fermented by stacking or packing, and different fermentation methods are adopted according to the state of tobacco leaves (Wang et al., 2009). Common fermentation media are mainly divided into microorganisms, aromatic substances, natural extracts, functional enzyme preparations, flavors and spices, etc., (Huang et al., 2013). In the process of fermentation, the medium participates in the metabolism of microorganisms and is transformed into aroma components or other substances that can improve the quality of tobacco leaves (Han et al., 1997; Zhang et al., 2021). Li Ning screened and isolated a strain of *Bacillus cereus* from the surface of cigar tobacco, prepared bacterial agent and added it to cigar tobacco for fermentation, which could significantly improve the quality of tobacco leaves (Li et al., 2009). Feng Yingjie added chlorogenic acid to cigar tobacco leaves to enhance flavor and improve quality (Feng, 2021). Xu Shijie explored the effect of feed liquid on the quality of artificially fermented cigar nightshade garment, and the study showed that the addition of feed liquid could effectively improve the chemical composition of tobacco leaves and improve the quality of tobacco leaves (Xu, 2016). Previous studies have shown that adding medium fermentation to tobacco leaves can effectively improve the quality of tobacco leaves, but the current studies are mostly focused on the agricultural fermentation stage, and rarely reported in the industrial fermentation stage (Zhang, 2022). China Tobacco Sichuan Greatwall Cigar Factory improved the technology on the basis of traditional fermentation technology and added liquid medium in the industrial fermentation stage (Li et al., 2016). Through sensory evaluation, it was found that the method played an important role in improving the quality of tobacco leaves.

This study mainly focuses on the research and analysis of the fermentation process of the factory adding feed liquid medium, and explores the effect of feed liquid on the main chemical components of filler tobacco leaves and the diversity of surface microorganisms. The raw material quality of domestic cigar and tobacco leaves is used as a technical reserve.

## 2 Materials and methods

### 2.1 Materials and instruments

#### 2.1.1 Cigar leaves

Frogstrips YA 14 of Brazilian original tobacco drying strips, supplied by China Tobacco Sichuan Great Wall Cigar Factory. Sodium hydroxide (sheet, AR), boric acid (AR), methyl Red, bromocresol Green, copper sulfate pentahydrate (AR), potassium sulfate (AR), concentrated sulfuric acid (98%), methanol, crystal sodium acetate, petroleum ether, chromatographic grade n-hexane, all purchased from Sinophosphora Chemical Reagent Co., LTD. The fermentation medium liquid is provided by China Tobacco Sichuan Great Wall Cigar Factory, the main ingredients include glutinous rice water, bulbus fritillariae cirrhosae, loquat syrup and other natural substances.

#### 2.1.2 Medium

*Fritillaria cirrhosa* extract 3 g, loquat extract 10 g, glutinous rice wine 20 g, dissolved in water to 100 ml, the initial medium pH = 6.2. In cigar tobacco leaves, every 100 g of cigar tobacco leaves added 10 g of formulated medium.

EL204 Electronic balance, Mettler Toledo Instruments (Shanghai) Co., LTD. Agilent High Performance Liquid chromatograph, Agilent; Automatic fat analyzer, Haineng Instrument Co., LTD. Ps-10 ultrasonic cleaning machine, Dongguan Jiekang Ultrasonic Equipment Co., LTD. SH420F Graphite digestion Instrument, Haineng Scientific Instrument Co., LTD. Haineng K9860 automatic Kjeldahl nitrogen analyzer, Haineng Scientific Instrument Co., LTD. Nexis GC-2030 Gas Chromatograph, Shimadzu, Japan.

### 2.2 Sample preparation and testing

#### 2.2.1 Fermention

**FIGURE 1:**
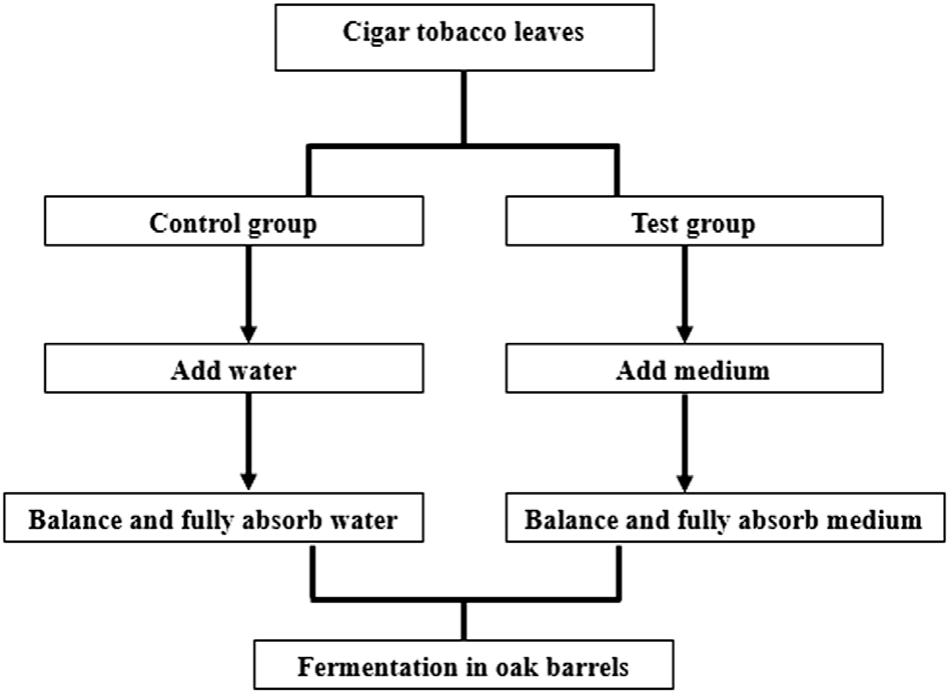
The fermentation steps of cigar tobacco leaves.

Loosen a handful of cigar tobacco leaves, spread flat on the table, using electronic sprayer medium evenly sprayed on the surface of the leaves. After spraying half, turn the tobacco leaves over and continue to spray the other side. After spraying, hang the tobacco leaves until the medium is fully absorbed by the tobacco leaves, and naturally absorb the medium After the medium is naturally absorbed, it is loaded into an oak barrel for fermentation. The cigar tobacco leaves in each oak barrel should not exceed 20 kg. Close the bucket lid. The oak barrels were placed in the fermentation room, the temperature of the fermentation room was controlled at 40°C, and the fermentation was carried out for 8 days. During the fermentation process, sampling was carried out on the 0th, 2nd, 4th, 6th and 8th days. We will generally take five positions of the cigar tobacco leaves, which are the four corners and the central position of the oak barrel, the five positions of the cigar tobacco leaves are ground and mixed together for testing. Detailed steps are shown in Figure 1.

#### 2.2.2 Determination of protein content

The protein content was determined with reference to the industry standard YC/T 249-2008 “Continuous Flow Method for the Determination of Protein in Tobacco and Tobacco Products” (Sha, 2020).

#### 2.2.3 Amino acid content determination

The amino acid content was determined with reference to the industry standard YC/T 448-2012 “Determination of Free Amino Acids in Tobacco and Tobacco Products by Ion Chromatography-Integral Pulse Amperometric Method” (Wang, 2017).

#### 2.2.4 Determination of petroleum ether extract content

The content of petroleum ether extract was determined with reference to the industry standard YC/T 176-2003 “Determination of Free Amino Acids in Tobacco and Tobacco Products by Ion Chromatography-Integral Pulse Amperometric Method” (Ding, 2014).

#### 2.2.5 Determination of fatty acid composition

The fatty acid content in tobacco leaf samples was determined by methylesterification-gas chromatography. The methylated samples were loaded on a Shimadzu gas chromatograph. Chromatographic conditions: The chromatographic column was Thermo Fisher Trace TR-FAME, 60 m × 0.25 mm × 0.25 μm; the temperature program was 130°C for 3 min, then increased to 200°C at 5°C/min and retained for 10 min, and then at 2°C/min to 220°C, hold for 3 min; the column flow rate is 1.8 ml/min (Deng, 2021).

#### 2.2.6 Determination of main aroma substances

##### 2.2.6.1 Sample pretreatment

Weigh about 1 g of the pulverized tobacco leaf sample and place it in a 22 ml headspace bottle, seal it with the headspace bottle cap, and extract the sample with a manual solid-phase microextraction device. An automated headspace solid-phase microextraction (HS-SPME) device equipped with 50/30 μm DVB/CAR/PDMS fibers was exposed to the headspace of the vial, and the extraction was performed at a needle speed of 20 mm/s for 30 min at 60°C. After extraction, desorption was performed on a GC analyzer in splitless mode at 250°C for 1 min, and volatiles were determined by GC-MS (Yan, 2019).

##### 2.2.6.2 GC conditions

RTX-waxMS column, flow rate 1.0 ml/min, helium as carrier gas, injection port temperature 250°C. The temperature programming conditions are as follows: hold at 60°C for 2 min, first increase to 110°C at 10°C/min, then increase to 150°C at 3°C/min, and finally increase to 230°C at 15°C/min, and hold for 20 min.

##### 2.2.6.3 MS conditions

Electron impact ion source (EI), ion source temperature 230°C, quadrupole temperature 150°C, transfer line temperature 230°C; electron energy: 70 eV, electron multiplier voltage: 1500 V; scan mass range from 45 to 350 m/z.

The identification of volatile compounds was based on the comparative analysis of the computer spectral library WILEY 8.0 and NIST 14. Only the identification results with positive and negative similarity greater than 800 were reported, and the relative content was calculated by the area normalization method.

#### 2.2.7 Determination of aroma precursors (polyphenols, glycosides)

Determination of aroma precursor substances such as polyphenols and glycosides in cigar wicks by liquid chromatography-mass spectrometry (Wu, 2012). The specific methods are Table 1 as follows:

(1) Sample pretreatment: Accurately weigh 1 g of cigarette sample (passed through 80 mesh sieve) in a conical flask, add 50 ml of methanol solution containing 2 µg/ml internal standard for ultrasonic extraction for 1 h, let stand, take 1 ml of supernatant and pass 0.22 µm The filter membrane was analyzed by UPLC-MS/MS.
(2) UPLC-MS/MS analysis: ACQUITYTMBET C18 column (1.7 µm, 2.1 mm × 100 mm, WATERS, United States), column temperature 40°C; sample chamber temperature 4°C; injection volume 1 µl; mobile phase A: methanol; B: 0.1% ammonium acetate monoacetate (10 mmol/L) aqueous solution; the flow rate is 0.4 ml/min, gradient elution, its parameters are shown in the table: 0–2 min, 10%A linear change to 100%A, and keep for 4 min; 6–7 min, 100%–10% A, step gradient (Tables 2–4).
(3) Mass spectrometry electrospray negative ion mode: electrospray voltage, −4500 V; ion source temperature (TEM); 500°C; ion source auxiliary gas 1, 60.0 kpa; ion source auxiliary gas 2, 60.0 kpa.

**TABLE 1:** Gradient elution parameter.

Time (min)	Mobile phase A%	Mobile phase B%	Curve
0	10	90	
2	100	0	6
6	100	0	1
7	10	90	1

**TABLE 2:** Effects of fermentation medium on amino acid composition of cigar leaf.

Amino acid (mg/g)	Control group					Test group				
	0 d	2 d	4 d	6 d	8 d	0 d	2 d	4 d	6 d	8 d
Asp	1.17 ± 0.02	1.25 ± 0.10	1.27 ± 0.03	1.08 ± 0.12	1.16 ± 0.0 3	1.26 ± 0.03	1.28 ± 0.21	0.97 ± 0.10	1.20 ± 0.29	1.17 ± 0.14
Glu	1.38 ± 0.01	1.49 ± 0.01	1.36 ± 0.04	1.42 ± 0.12	1.38 ± 0.13	1.40 ± 0.14	1.64 ± 0.21	1.29 ± 0.21	1.48 ± 0.03	1.29 ± 0.27
Ser	0.43 ± 0.00	0.03 ± 0.00	0.40 ± 0.01	0.07 ± 0.00	0.37 ± 0.01	0.42 ± 0.00	0.44 ± 0.01	0.35 ± 0.00	0.40 ± 0.01	0.03 ± 0.01
His	0.19 ± 0.01	0.20 ± 0.01	0.18 ± 0.00	0.01 ± 0.00	0.18 ± 0.00	0.21 ± 0.00	0.19 ± 0.01	0.17 ± 0.00	0.16 ± 0.00	0.01 ± 0.00
Gly	0.65 ± 0.02	0.69 ± 0.00	0.64 ± 0.01	0.52 ± 0.01	0.62 ± 0.02	0.68 ± 0.10	0.69 ± 0.11	0.55 ± 0.01	0.66 ± 0.00	0.61 ± 0.01
Thr	0.45 ± 0.00	0.46 ± 0.01	0.43 ± 0.01	0.35 ± 0.01	0.40 ± 0.01	0.46 ± 0.01	0.47 ± 0.01	0.37 ± 0.01	0.42 ± 0.01	0.42 ± 0.01
Arg	0.37 ± 0.01	0.40 ± 0.01	0.35 ± 0.01	0.81 ± 0.02	0.33 ± 0.00	4.24 ± 0.03	0.39 ± 0.01	0.27 ± 0.00	0.39 ± 0.00	0.48 ± 0.00
Ala	0.53 ± 0.01	0.55 ± 0.01	0.51 ± 0.00	0.05 ± 0.00	0.49 ± 0.00	0.55 ± 0.02	0.55 ± 0.01	0.43 ± 0.00	0.55 ± 0.01	0.51 ± 0.00
Tyr	0.27 ± 0.00	0.28 ± 0.00	0.26 ± 0.01	0.15 ± 0.00	0.21 ± 0.00	0.25 ± 0.03	0.29 ± 0.01	0.21 ± 0.01	0.26 ± 0.01	0.25 ± 0.01
Cys	0.00 ± 0.00	0.00 ± 0.00	0.00 ± 0.00	0.01 ± 0.00	0.01 ± 0.00	0.01 ± 0.00	0.01 ± 0.00	0.01 ± 0.00	0.00 ± 0.00	0.06 ± 0.01
Val	0.60 ± 0.02	0.62 ± 0.01	0.57 ± 0.01	0.47 ± 0.02	0.57 ± 0.01	0.63 ± 0.01	0.64 ± 0.01	0.48 ± 0.02	0.64 ± 0.00	0.56 ± 0.00
Met	0.13 ± 0.01	0.13 ± 0.01	0.11 ± 0.00	0.11 ± 0.00	0.12 ± 0.00	0.13 ± 0.00	0.13 ± 0.00	0.10 ± 0.00	0.12 ± 0.00	0.12 ± 0.01
Phe	0.50 ± 0.01	0.54 ± 0.02	0.47 ± 0.00	0.40 ± 0.00	0.48 ± 0.02	0.52 ± 0.00	0.53 ± 0.01	0.42 ± 0.01	0.52 ± 0.01	0.47 ± 0.00
Ile	0.44 ± 0.01	0.47 ± 0.01	0.42 ± 0.00	0.33 ± 0.02	0.41 ± 0.01	0.47 ± 0.01	0.46 ± 0.02	0.36 ± 0.00	0.47 ± 0.01	0.41 ± 0.01
Leu	0.69 ± 0.01	0.73 ± 0.01	0.64 ± 0.01	0.53 ± 0.01	0.64 ± 0.02	0.73 ± 0.02	0.73 ± 0.02	0.57 ± 0.01	0.72 ± 0.01	0.66 ± 0.00
Lys	0.29 ± 0.00	0.27 ± 0.00	0.29 ± 0.00	0.20 ± 0.02	0.24 ± 0.00	0.31 ± 0.02	0.34 ± 0.01	0.23 ± 0.00	0.33 ± 0.01	0.29 ± 0.00
Pro	0.47 ± 0.00	0.57 ± 0.00	0.52 ± 0.00	0.44 ± 0.01	0.56 ± 0.01	0.56 ± 0.00	0.57 ± 0.02	0.48 ± 0.02	0.62 ± 0.02	0.43 ± 0.01
Total	8.57 ± 0.14	8.69 ± 0.21	8.44 ± 0.14	6.95 ± 0.36	8.17 ± 0.27	12.82 ± 0.42	9.35 ± 0.68	7.25 ± 0.39	8.93 ± 0.42	7.76 ± 0.49

**TABLE 3:** *T*-test results of total major amino acids.

Fermentation time	*P*
0 d (Control group vs. test group)	2.86e-01
2 d (Control group vs. test group)	1.40e-01
4 d (Control group vs. test group)	4.86e-04
6 d (Control group vs. test group)	1.78e-02
8 d (Control group vs. test group)	3.83e-01

**TABLE 4:** Fatty acid composition of cigar tobacco leaves after fermentation.

	Control group					Test group				
Fattyacid/%	0 d	2 d	4 d	6 d	8 d	0 d	2 d	4 d	6 d	8 d
C6:0	0.69 ± 0.28	0.47 ± 0.26	0.27 ± 0.02	0.53 ± 0.01	2.35 ± 0.25	2.16 ± 0.00	0.53 ± 0.05	0.21 ± 0.02	0.58 ± 0.62	3.61 ± 1.03
C8:0	1.92 ± 1.48	0.44 ± 0.10	0.80 ± 0.62	1.73 ± 1.50	nd	0.56 ± 0.09	0.58 ± 0.00	0.73 ± 0.73	0.62 ± 0.26	0.65 ± 0.37
C10:0	0.68 ± 0.28	0.10 ± 0.15	2.09 ± 1.27	0.98 ± 0.63	nd	nd	0.66 ± 0.07	1.28 ± 1.27	3.29 ± 3.45	0.22 ± 0.31
C11:0	0.67 ± 0.05	0.86 ± 0.42	0.60 ± 0.03	1.10 ± 0.85	0.32 ± 0.46	1.05 ± 0.22	0.75 ± 0.30	1.91 ± 1.07	1.34 ± 0.78	0.94 ± 1.33
C12:0	0.24 ± 0.34	0.88 ± 0.07	1.29 ± 1.37	2.65 ± 1.12	0.28 ± 0.40	0.25 ± 0.35	0.70 ± 0.65	1.09 ± 0.52	1.32 ± 0.31	0.29 ± 0.41
C13:0	0.63 ± 0.12	0.74 ± 0.18	1.50 ± 1.45	1.75 ± 1.42	0.82 ± 0.17	1.54 ± 0.08	1.11 ± 0.53	1.74 ± 0.32	1.13 ± 0.45	1.37 ± 1.25
C14:0	1.20 ± 0.28	3.99 ± 1.40	5.54 ± 3.01	3.15 ± 0.52	2.23 ± 0.06	1.54 ± 0.23	3.58 ± 0.96	3.52 ± 0.21	3.26 ± 0.59	1.67 ± 0.59
C14:1	1.07 ± 0.53	0.85 ± 0.06	1.28 ± 0.38	1.16 ± 0.44	nd	0.43 ± 0.08	2.82 ± 1.51	0	1.05 ± 0.79	0.55 ± 0.11
C15:0	1.11 ± 0.17	2.30 ± 0.26	4.88 ± 2.04	4.85 ± 1.28	1.19 ± 0.15	1.21 ± 0.07	4.62 ± 0.74	4.00 ± 0.50	2.55 ± 1.51	1.04 ± 0.34
C15:1	1.14 ± 0.15	1.93 ± 1.06	2.82 ± 0.92	1.74 ± 1.11	1.01 ± 0.12	0.55 ± 0.17	1.17 ± 0.65	0.82 ± 0.36	2.16 ± 1.94	0.71 ± 0.11
C16:0	25.49 ± 3.21	26.29 ± 5.08	24.81 ± 5.53	23.53 ± 1.52	26.11 ± 5.64	20.94 ± 4.08	24.67 ± 0.37	21.42 ± 1.17	25.54 ± 2.74	21.47 ± 2.62
C16:1	3.70 ± 0.31	1.37 ± 0.73	2.00 ± 0.67	0.80 ± 0.39	1.19 ± 0.66	2.27 ± 2.45	1.06 ± 0.23	2.34 ± 0.69	1.42 ± 0.05	1.87 ± 1.63
C17:1	6.96 ± 1.89	3.81 ± 4.41	2.83 ± 3.56	4.07 ± 0.81	0.49 ± 0.70	0.44 ± 0.02	3.61 ± 1.55	1.76 ± 0.25	2.72 ± 1.52	0.24 ± 0.34
C18:0	6.26 ± 4.99	8.51 ± 0.01	7.57 ± 2.13	8.38 ± 0.34	7.27 ± 2.23	5.38 ± 0.88	9.56 ± 1.32	6.84 ± 3.35	9.16 ± 3.17	5.68 ± 0.89
C18:1(T)	0.90 ± 0.51	1.03 ± 0.60	0.96 ± 0.48	1.62 ± 1.28	nd	nd	1.00 ± 0.04	0.80 ± 0.61	1.11 ± 0.64	nd
C18:1	nd	12.04 ± 2.33	8.34 ± 0.02	8.54 ± 0.13	8.29 ± 0.05	7.69 ± 0.77	10.06 ± 0.34	11.54 ± 0.12	8.38 ± 0.07	8.55 ± 1.71
C18:2(T)	nd	0.49 ± 0.29	0.82 ± 0.71	0.81 ± 0.04	1.10 ± 0.39	2.07 ± 0.20	0.88 ± 0.01	1.13 ± 0.10	0.95 ± 0.11	0.64 ± 0.33
C18:2	7.36 ± 0.70	7.37 ± 1.17	7.32 ± 1.72	7.70 ± 0.16	5.19 ± 0.39	7.36 ± 1.04	8.10 ± 1.48	9.96 ± 0.46	6.59 ± 1.31	6.97 ± 1.51
C20:0	2.12 ± 1.79	0.39 ± 0.02	0.69 ± 0.18	1.59 ± 0.04	3.80 ± 1.55	3.18 ± 3.64	0.53 ± 0.00	1.29 ± 0.68	1.79 ± 0.19	0.58 ± 0.14
C18:3(γ)	6.41 ± 1.44	10.46 ± 2.08	6.65 ± 1.22	5.85 ± 1.09	3.05 ± 0.09	4.84 ± 0.81	7.52 ± 0.35	9.98 ± 1.55	6.35 ± 0.56	4.14 ± 1.60
C20:1	2.00 ± 1.05	1.00 ± 0.71	2.02 ± 0.87	1.02 ± 0.10	2.04 ± 0.64	1.77 ± 0.45	2.82 ± 0.53	0.87 ± 0.25	1.49 ± 1.44	1.12 ± 0.25
C18:3(α)	0.44 ± 0.62	0.78 ± 0.25	0.83 ± 0.60	1.08 ± 0.92	0.85 ± 0.33	0.38 ± 0.04	0.42 ± 0.22	0.60 ± 0.38	0.94 ± 0.08	nd
C21:0	nd	1.38 ± 0.15	0.77 ± 0.70	1.13 ± 0.37	0.40 ± 0.56	1.96 ± 0.30	0.51 ± 0.26	1.07 ± 0.33	0.53 ± 0.74	2.02 ± 2.18
C20:2	0.83 ± 0.44	0.61 ± 0.14	0.86 ± 0.55	0.85 ± 0.84	0.66 ± 0.94	1.24 ± 1.04	0.29 ± 0.02	1.03 ± 0.92	0.66 ± 0.02	0.77 ± 0.11
C22:0	3.01 ± 1.24	0.72 ± 0.30	0.94 ± 0.92	0.69 ± 0.42	4.60 ± 1.44	2.20 ± 1.86	1.05 ± 0.46	1.09 ± 0.69	0.91 ± 0.12	4.01 ± 1.93
cis-8, 11,14-C22:1	0.40 ± 0.56	0.66 ± 0.21	0.82 ± 0.49	1.93 ± 1.39	nd	nd	1.55 ± 0.35	1.13 ± 0.65	1.81 ± 0.26	0.27 ± 0.38
Cis-11, 14,17-C20:3	nd	0.67 ± 0.27	0.83 ± 0.33	0.96 ± 0.41	nd	nd	0.41 ± 0.17	1.36 ± 0.32	1.40 ± 0.19	nd
C20:4	0.76 ± 0.02	0.38 ± 0.19	1.21 ± 0.99	1.78 ± 0.55	0.94 ± 0.24	0.86 ± 0.40	0.66 ± 0.00	2.35 ± 1.58	2.17 ± 2.33	1.12 ± 0.60
C23:0	0.95 ± 0.40	0.92 ± 0.31	1.62 ± 0.38	1.86 ± 0.42	1.08 ± 0.80	0.47 ± 0.04	1.49 ± 0.59	0.55 ± 0.10	1.48 ± 1.03	0.55 ± 0.01
C22:2	0.66 ± 0.28	0.89 ± 0.20	1.97 ± 2.22	0.61 ± 0.17	nd	0.62 ± 0.09	1.50 ± 1.16	1.48 ± 1.34	1.00 ± 0.98	0.39 ± 0.55
C24:0	nd	1.12 ± 0.07	0.59 ± 0.30	0.96 ± 0.23	nd	0.52 ± 0.29	0.89 ± 0.03	0.80 ± 0.84	0.51 ± 0.17	nd
C20:5	20.33 ± 11.60	1.59 ± 0.99	0.51 ± 0.14	1.45 ± 0.21	23.06 ± 9.28	23.85 ± 10.67	1.00 ± 0.85	1.33 ± 1.02	0.83 ± 0.38	26.20 ± 3.94
C24:1	0.60 ± 0.20	0.80 ± 0.20	0.85 ± 0.26	1.11 ± 0.04	0.94 ± 0.23	1.69 ± 1.17	0.85 ± 0.71	1.07 ± 0.04	0.97 ± 0.34	0.54 ± 0.20

#### 2.2.8 Content determination of free fragrance components

For cigar tobacco leaf samples, the free aroma components were determined by the solvent extraction method (Liu, 1998). The main methods Table 2 are as follows:

(1) Connect a 1000 ml round-bottomed flask containing 20 g of smoke sample, 2 g of citric acid and 600 ml of distilled water to one end of the simultaneous distillation and extraction device, and use a constant temperature electric heating mantle for heating; the other end of the device is connected to 40 ml of dichloride A 250 ml round-bottomed flask containing methane and 1 ml of internal standard, the end of the flask was heated in a constant temperature pot with a water bath temperature of 60°C, and 20 g of anhydrous sodium sulfate was added to dry the organic phase after distillation and extraction for 2.5 h, and then the organic phase was dried in a water bath at 60°C and concentrated to about 1 ml. The analytical samples prepared by pretreatment were identified by GC/MS and NISD library search.
(2) GC-MS analysis conditions Chromatographic column: PE-5MS capillary column (30 m × 0.25 mm, 0.25 μm, American PE company; injection port temperature: 250°C; temperature program: 50°C for 2 min, and then increased to 250°C at a rate of 4°C/min; carrier gas: helium (He); flow rate: 1 ml/min; injection volume: 1 μl; GC-MS analysis conditions: PE-5MS capillary column (30 m × 0.25 mm × 0.25 µm); Inlet temperature: 250°C; Program temperature: 50°C for 2 min, 50-250 (4°C/min); Carrier gas He; Flow rate: 1 ml/min; Injection volume: 1 µl; Split ratio: 30: 1. Ion source: EI; ionization energy: 70 eV; ion source temperature: 170°C; transfer line temperature: 250°C;

#### 2.2.9 Sensory evaluation

The fermented cigar tobacco leaves were rolled into cigars with 130 mm in length and 30 mm in circumference. Select 5-7 qualified personnel, distribute the score sheet, evaluate the product without interference, and finally use the average score to evaluate (Shen, 2011).

#### 2.2.10 Determination of microbial diversity

Total bacterial DNA was extracted according to the instructions of E.Z.N.A.^®^ soil DNA kit (Omega Bio-tek, Norcross, GA, United States), the quality of DNA extracted was detected by 1% agarose gel electrophoresis, and the DNA concentration and purity were determined by Meder et al. (2011). The V3∼V4 region of 16S rRNA gene was amplified by PCR using 338F (5′-ACT​CCT​ACG​GGA​GGC​AGC​AG-3′) and 806R (5′-GACTACHVGGGTTWTCTAAT-3′) (Langille, 2013). PCR products were recovered by 2% agarose gel, purified by AxyPrep DNA Gel Extraction Kit (Axygen Biosciences, Union City, CA, United States), and detected and quantified by Quantus™ Fluorometer. Amplified sequence libraries were established using the NEXTFLEX Rapid DNA-Seq Kit (New England Biolabs Inc., Ipswich, 127 MA, United States), followed by high-throughput sequencing using the Illumina Miseq PE300 sequencing platform (Illumina Corporation, San Diego, United States). The original sequences were spliced by FLASH software, and the sequences were OTU clustered by USEARCH software (version 7.0) and chimera sequences were eliminated (Nguyen, 2016). The dilution curve, species composition and abundance distribution table were obtained by calculation, and the functional genes of the sample colonies were predicted by the PICRUSt2 method.

### 2.3 Data analysis

The statistical significance of the data was tested by Student’s method in JMP 13 statistical software, and the data was plotted by Origin software. Paired *t*-test was used to analyze and compare relevant experimental data.

## 3 Result

### 3.1 Effects of fermentation medium on protein content of cigar tobacco leaves

**FIGURE 2:**
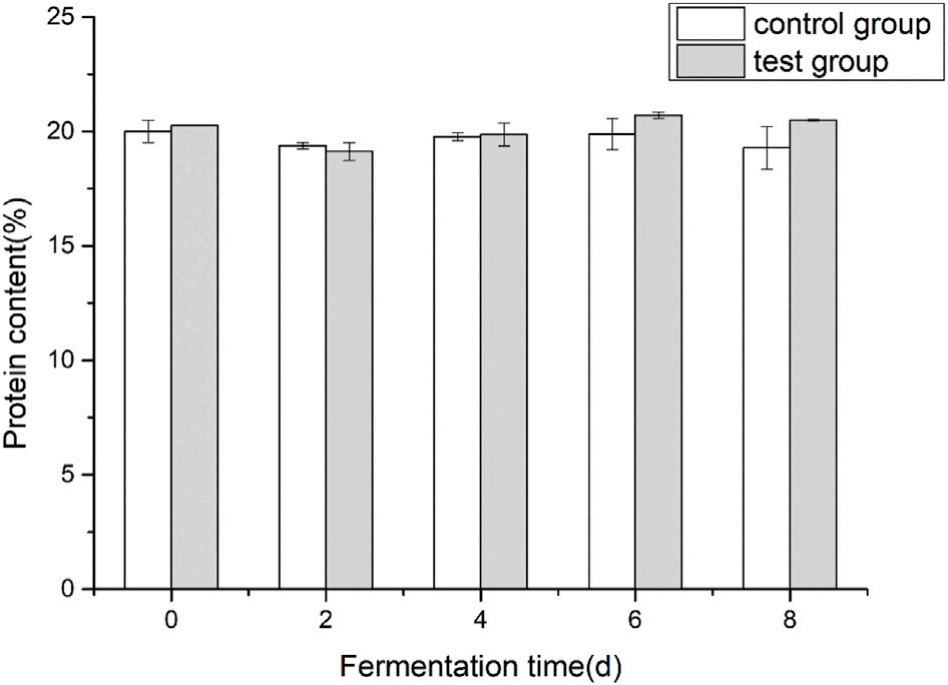
Effect of fermentation on the protein contents of cigar tobacco leaves.

Comparing the control group and the test group, it can be seen from Figure 2 that the overall change law of the protein content of cigar tobacco leaves during the fermentation process is not significant. The protein content was 19.29% ± 0.93% and 20.49 ± 0.03%. The protein content of the experimental group was higher than that of the control group at the later stage of fermentation.

### 3.2 Effect of fermentation medium on amino acid composition of cigar tobacco leaves

SPSS software was used for analysis according to paired *t* test, and the critical level was set to *p* = 0.05. The data analysis results are shown in Table 3. During the fermentation process, on the 4th and 6th day, the *p*-values of the control group and the feed-added test group were both less than 0.05, and the difference was significant. On the 8th day after the fermentation, there was no significant difference in the total amount of amino acids between the two groups. On the 6th day of fermentation, the total amount of amino acids in the feeding experimental group was higher than that in the control group. Considering the changes in the total amino acid, with the extension of the fermentation time, the total amino acid in the feeding experimental group decreased rapidly, which may The sensory quality of cigar tobacco leaves is affected, and the end time of fermentation can be appropriately advanced.

### 3.3 Effects of fermentation medium on the content of petroleum ether extract in cigar tobacco leaves

**FIGURE 3:**
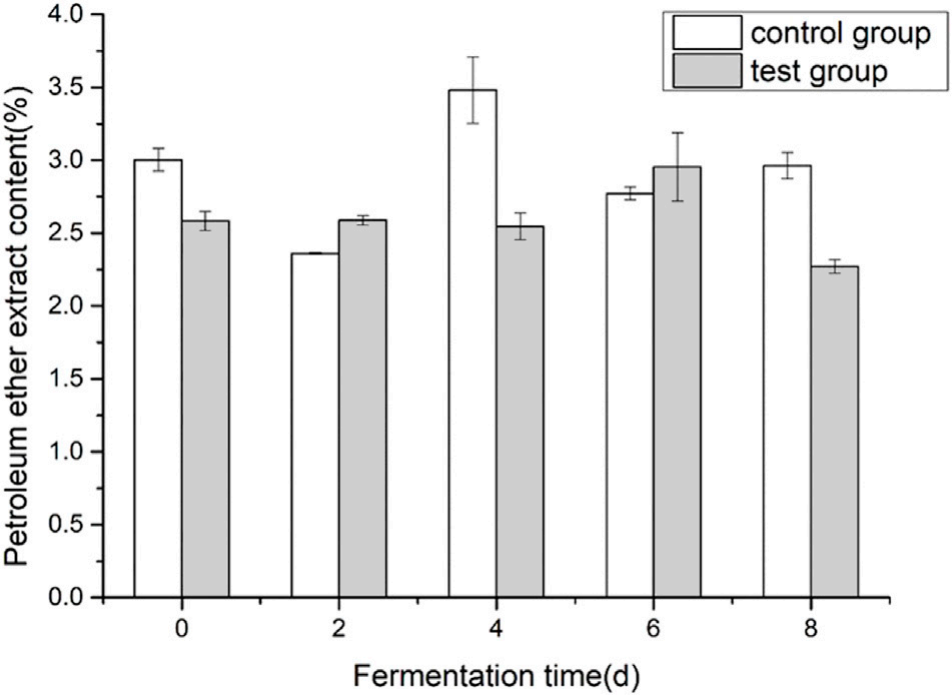
Effects of fermentation medium on the content of petroleum ether extract from cigar tobacco.

Cigar tobacco leaf petroleum ether extract is a mixture obtained by using petroleum ether as a solvent to extract tobacco leaves. The main components are volatile oils, resins, oils, fatty acids, waxes, lipids, pigments, and other substances. The petroleum ether extract in tobacco leaves is mainly related to the aroma of tobacco. During the alcoholization process of tobacco leaves, it is transformed and decomposed to form aroma substances. Therefore, petroleum ether extract is usually used to measure the quality and aroma of tobacco leaves. Important indicators. As shown in Figure 3, during the fermentation process of cigar tobacco leaves, the overall trend of the content of petroleum ether extract showed that it first increased and then decreased. The content of petroleum ether extract before and after fermentation in the control group did not change much, decreased by 1.35%, and the addition of the experimental group decreased significantly, decreased by 12.4%. The addition of fermentation medium reduced the content of petroleum ether extract to some extent.

### 3.4 Effects of fermentation medium on fatty acid composition of cigar tobacco leaves

A total of 34 fatty acids were detected in cigar tobacco leaves, as shown in Table 4. The fatty acid content changed significantly during the fermentation process. The main representative fatty acids were C6:0 (caprylic acid), C8:0 (caprylic acid monoglyceride), C14:0 (myristic acid), C11:0 (undecanoic acid), C16:0 (palmitic acid), C17:1, C18:0 (stearic acid), C18:3 (gamma), C20: 0 (arachidic acid), C20:1 (eicosaenoic acid), C20:5 (eicosapentaenoic acid), and C22:0 (docosanoic acid triglyceride). After the tobacco leaves were fermented, the fatty acid composition of the control group and the feed-added test group showed the same change rule. The relative content of unsaturated fatty acids in the test group decreased from 34.68% to 35.19%–25.88% and 33.46%, respectively. Unsaturated fatty acids in the control group decreased significantly.

### 3.5 Effects of fermentation medium on main compounds in cigar tobacco leaves

**FIGURE 4:**
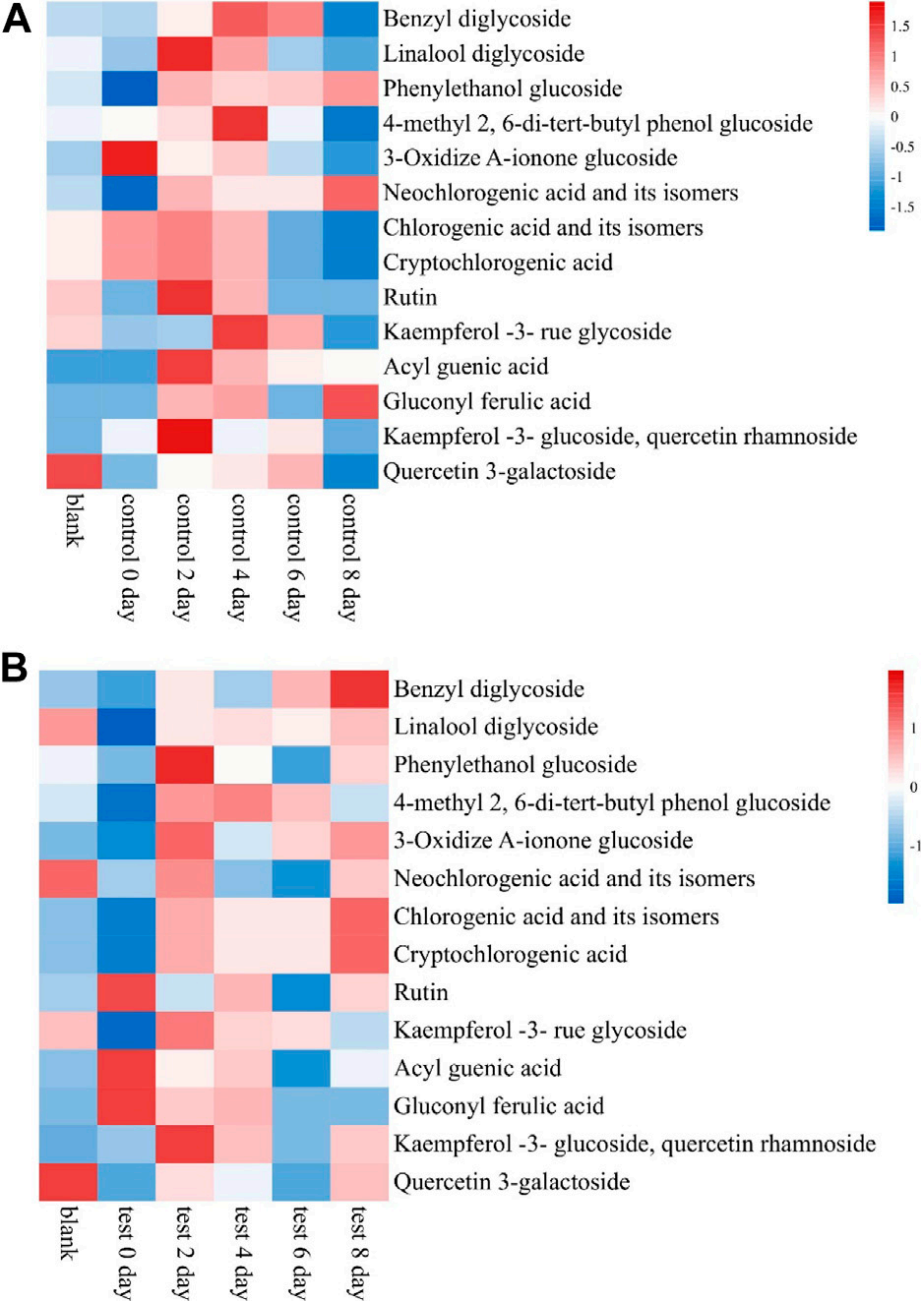
Effects of medium fermentation on aroma precursors of cigar tobacco. Note: **(A)** is the control group, and **(B)** is the test group.

About 65 compounds were identified in Brazilian Frogstrips YA14 tobacco leaves before and after fermentation. Fermentation affects the distribution of aroma components in tobacco leaves, which is beneficial to improve the aroma components of tobacco leaves, such as 3-methylvaleric acid, phenethyl alcohol, solanone, etc., and reduce nicotine and diene. The relative content of nicotine, etc., is shown in Table 5. As can be seen from Figure 4, from the results of sampling detection and analysis at different times, with the extension of fermentation time, the overall aroma precursor substances in tobacco leaves increased first and then decreased, which is consistent with the changes in amino acids above. From this, it can be inferred that too long fermentation time will affect the content of aroma components in cigar leaves, moderate fermentation is beneficial to improve the quality of tobacco leaves, and excessive fermentation will damage the quality of tobacco leaves. After the fermentation, the content of aroma precursor substances in the control group was lower than that in the addition test group, which was consistent with the sensory evaluation results. The addition of medium fermentation could significantly increase the aroma precursor substances in tobacco leaves.

**TABLE 5:** Brazil Frogstrips YA14 Main compounds change during smoke core fermentation.

Serial number	Keep time	CAS	Compound name	Control group					Test group				
				0 d	2 d	4 d	6 d	8 d	0 d	2 d	4 d	6 d	8 d
1	2.13	75-50-3	Trimethylamine	nd	0.03	nd	nd	nd	nd	nd	nd	nd	nd
2	2.64	13475-82-6	2,2,4,6,6-Pentamethylheptane	23.00	0.16	nd	0.23	nd	nd	0.23	0.14	0.17	0.25
3	2.99	4390-04-9	Isohexadecane	0.03	0.02	nd	0.04	nd	nd	0.03	nd	0.03	nd
4	2.99	62183-79-3	2,2,4,4-Tetrahydromethyloctane	0.03	0.02	0.02	0.04	nd	0.02	0.03	0.01	0.03	0.03
5	4.55	3913-02-8	2-Butyloctanol	0.01	0.04	0.01	nd	0.03	0.01	0.01	nd	nd	nd
6	4.86	629-50-5	Tridecane	nd	0.04	nd	0.05	nd	nd	nd	nd	nd	nd
7	6.73	110-93-0	Methylheptenone	0.23	0.09	0.10	0.06	0.04	0.11	0.10	0.11	0.05	0.04
8	6.82	3891-98-3	2,6,10-Trimethyldodecane	0.02	0.04	nd	nd	0.05	nd	0.02	nd	nd	0.02
9	7.47	629-59-4	Tetradecane	0.12	0.10	0.10	0.10	0.09	0.08	0.12	0.08	0.10	0.13
10	8.01	629-92-5	n-Nadecane	nd	0.06	nd	nd	nd	nd	0.06	nd	nd	nd
11	8.37	64-19-7	Glacial acetic acid	0.07	nd	0.02	0.04	nd	nd	0.07	0.03	nd	0.17
12	8.57	102-27-2	N-ethyl-m-toluidine	0.09	0.07	0.07	0.06	0.06	0.04	0.06	0.07	0.06	0.05
13	9.73	100-52-7	Benzaldehyde	0.04	0.08	0.06	0.08	0.08	0.07	0.13	0.10	0.08	0.09
14	12.3	98-86-2	Acetophenone	0.18	0.20	0.18	0.23	0.20	0.18	0.18	0.17	0.20	nd
15	12.73	1073-11-6	4-Methyl-4-hydroxy-5-hexenoic acid-γ-lactone	0.06	0.06	0.06	0.07	0.08	0.05	0.15	0.06	0.08	nd
16	13.04	1604-34-8	Hexahydropseudoionone	0.56	0.50	0.40	0.70	0.70	0.40	0.56	0.44	0.47	nd
17	13.23	1125-21-9	4-oxoisophorone	0.11	0.13	0.16	0.14	0.12	0.13	0.22	0.12	0.13	0.19
18	14.09	54868-48-3	Solanone	0.78	0.69	0.81	0.84	0.78	0.81	0.92	0.78	0.74	0.81
19	14.64	100-54-9	3-cyanopyridine	0.13	0.11	0.09	0.10	0.11	0.05	0.08	0.08	0.11	0.08
20	14.87	14237-73-1	2-Hexadecene, 3,7,11,15-tetramethyl-, [R-[R*,R*-(E)]]-	nd	0.09	0.10	0.13	0.14	0.10	0.11	0.07	0.10	nd
21	15.37	20547-99-3	2,2,6-Trimethyl-1,4-cyclohexanedione	0.08	0.06	0.08	0.09	0.08	0.08	0.08	0.07	0.07	nd
22	16.13	105-43-1	3-Methylvaleric acid	0.09	0.08	0.19	0.29	0.10	0.17	0.38	0.14	0.24	0.46
23	16.73	1122-54-9	4-Acetylpyridine	1.76	2.06	1.82	2.02	2.56	1.82	1.76	2.05	2.16	2.32
24	17.02	17283-81-7	BETA-Dihydroionone	0.04	0.10	0.08	0.09	0.07	0.08	0.04	0.10	0.08	0.04
25	17.77	23950-04-1	2-(1-Methylpyrrolidin-2-yl)pyridine	0.01	0.07	50.22	0.02	0.13	0.02	0.02	50.50	0.15	0.18
26	17.96	54-11-5	Nicotine	71.95	70.90	65.19	72.03	70.08	72.61	69.12	72.83	69.02	67.14
27	19	60-12-8	Phenylethanol	0.18	0.08	0.12	0.13	0.13	0.12	0.18	0.14	0.15	0.18
28	19.48	61886-66-6	3-eicosyne	11.70	nd	11.31	10.67	11.32	11.68	12.99	10.90	12.92	13.17
29	19.54	102608-53-7	Phytol	11.70	12.50	11.31	10.67	11.32	11.68	12.99	10.90	12.92	13.17
30	19.61	79-77-6	Beta-ionone	0.08	nd	nd	0.08	0.09	0.07	nd	0.08	0.06	0.08
31	20.44	634-36-6	1,2,3-Trimethoxybenzene	0.08	0.06	0.06	0.08	0.08	0.06	0.08	0.07	0.04	0.07
32	20.48	NA	Phenyl 3,4-dimethylvalerate	0.05	nd	0.04	0.05	0.06	0.04	0.05	0.05	0.05	0.05
33	21.02	23267-57-4	4-[2,2,6-Trimethyl-7-oxabicyclo[4.1.0]heptan-1-yl]-3- buten-2-one	0.13	0.12	0.12	0.15	0.16	0.12	0.15	0.13	0.13	0.15
34	22.14	6443-69-2	3,4,5-Trimethoxytoluene	0.10	0.10	0.08	0.14	0.13	0.09	0.09	0.11	0.11	0.11
35	23.17	502-69-2	Phytone	0.32	0.45	0.29	0.35	0.36	0.29	0.31	0.28	0.41	0.31
36	23.97	532-12-7	Maxmin	0.64	0.89	0.83	0.65	0.87	0.81	0.67	0.83	0.86	0.75
37	24.23	NA	7-Hydroxy-2,2,6,7-tetramethylbicyclo[4,3,0]nonane			0.06	0.06	0.06	0.05	0.06	0.05	0.07	0.07
38	24.41	939-23-1	4-Phenylpyridine	0.05	0.06	0.05	0.05	0.06	0.05	0.05	0.05	0.05	0.06
39	24.95	96-76-4	2,4-Di-tert-butylphenol	0.12	nd	0.02	0.02	nd	0.02	0.12	0.10	0.06	nd
40	25.14	487-19-4	Dienenicotine, Nicotaine	2.12	2.86	1.93	1.76	3.20	2.05	1.54	2.24	2.04	1.81
41	25.31	17092-92-1	Dihydrokiwifruit lactone	0.50	0.57	0.51	0.56	0.58	0.52	0.54	0.54	0.56	0.59
42	25.42	77-67-8	Ethosuximide	0.06	0.07	0.06	0.07	0.04	0.07	0.08	0.07	0.08	0.08
43	25.49	1117-52-8	Farnesyl acetone	0.06	0.08	0.05	0.08	0.08	0.04	0.04	0.04	0.07	0.05
44	26.05	54060-30-9	m-aminophenylacetylene	0.02	nd	0.04	nd	nd	0.03	nd	nd	nd	nd
45	26.07	120-72-9	Indole	0.71	0.03	0.04	0.03	0.03	0.71	0.03	0.03	0.04	0.05
46	26.42	581-50-0	2,3′-bipyridine	0.61	0.96	0.79	0.79	0.99	0.79	0.61	0.77	0.96	0.92
47	27.57	150-86-7	Phytol	0.02	0.09	nd	0.03	0.04	0.02	0.03	0.04	0.07	0.04
48	30.96	486-56-6	Cotinine	0.02	0.02	0.02	0.01	0.03	0.02	0.02	0.02	0.03	0.02

### 3.6 Effect of fermentation medium on microbial diversity of cigar leaf surface

#### 3.6.1 Alpha diversity analysis

The Alpha diversity index of microorganisms in the samples of the control group and the test group is shown in Table 1. The diversity and richness of bacteria and fungi in the samples were assessed using Chao1, Shannon, Simpson, and Coverage indices in the Alpha diversity index. The microbial coverage index of all samples in the table is greater than 0.99, indicating that the sequence detection in the sample library is basically covered, and the sample sequencing can reflect the real situation of the sample. It can be seen from Table 6 that during the fermentation process, the shannon and Chao1 indices of the control group and the test group both increased first and then decreased, and the overall levels were higher than those before fermentation. The simpson index was negatively correlated with shannon. The levels were lower than those before fermentation, indicating that the species richness of tobacco leaves increased after fermentation. At the same time, it can be seen from the indices in the figure that the species richness and community diversity of the test group were slightly higher than those of the control group.

**TABLE 6:** Effects of fermentation medium on microbial diversity on the surface of cigar tobacco leaves.

Sample	Shannon	Simpson	Chao1	Coverage
Control group 0 d	0.39	0.86	94.00	0.99
Control group 2 d	1.33	0.41	116.00	0.99
Control group 4 d	1.66	0.42	300.08	0.99
Control group 6 d	2.01	0.21	184.38	0.99
Control group 8 d	1.16	0.42	167.33	0.99
Test group 0 d	1.14	0.56	183.64	0.99
Test group 2 d	3.24	0.07	326.00	0.99
Test group 4 d	1.14	0.45	211.56	0.99
Test group 6 d	1.69	0.37	161.61	0.99
Test group 8 d	1.29	0.38	178.66	0.99

#### 3.6.2 OTU analysis of microbial communities

**FIGURE 5:**
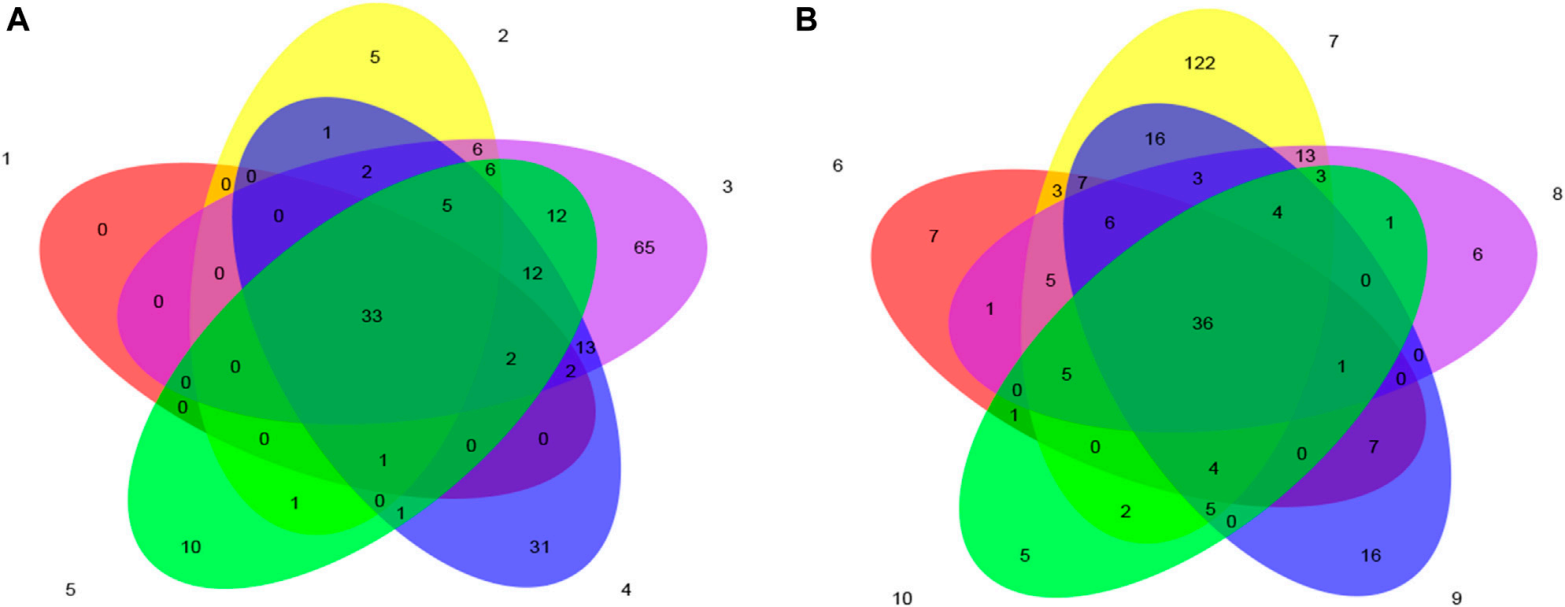
Venn diagram based on OTU of microbial community in cigar tobacco leaves. Note: **(A)** is the control group, and **(B)** is the test group.

The Venn diagram is used to represent the number of common and unique OTUs in the fermentation process of tobacco leaves, which can intuitively reflect the similarity and overlap of the number of OTUs. It can be seen from Figure 5 that the control group had the largest number of OTUs on the fourth day of fermentation, the experimental group had the largest number of OTUs on the second day of fermentation, the total number of OTUs in the control group was 33, and the number of OTUs in the experimental group was 36. During the process, the community structure similarity of the test group was higher.

#### 3.6.3 Microbial community structure analysis

**FIGURE 6:**
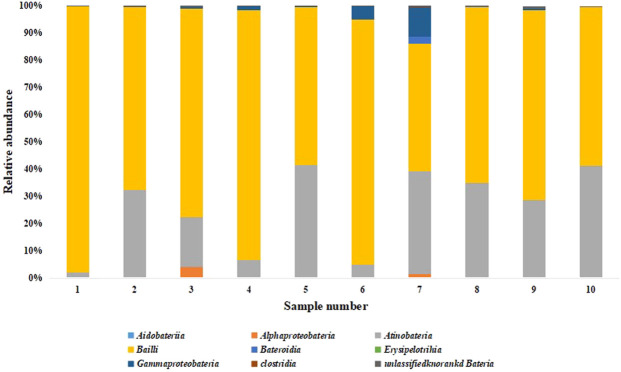
Relative abundance of bacterial phylum. Note: 1 to 5 were respectively added water and fermented for 0 d, 2 d, 4 d, 6 d, 8 d, 6-10 were followed by medium fermentation for 0 d, 2 d, 4 d, 6 d, 8 d.

A total of 5 bacterial phyla were detected at the phylum level, namely Proteobacteria, Actinobacteria, Bacteroidetes, Firmicutes and an unknown phylum. It can be seen from Figure 6 that Firmicutes has the highest relative abundance at different stages of the two groups of fermentation samples, followed by Actinobacteria.

**FIGURE 7:**
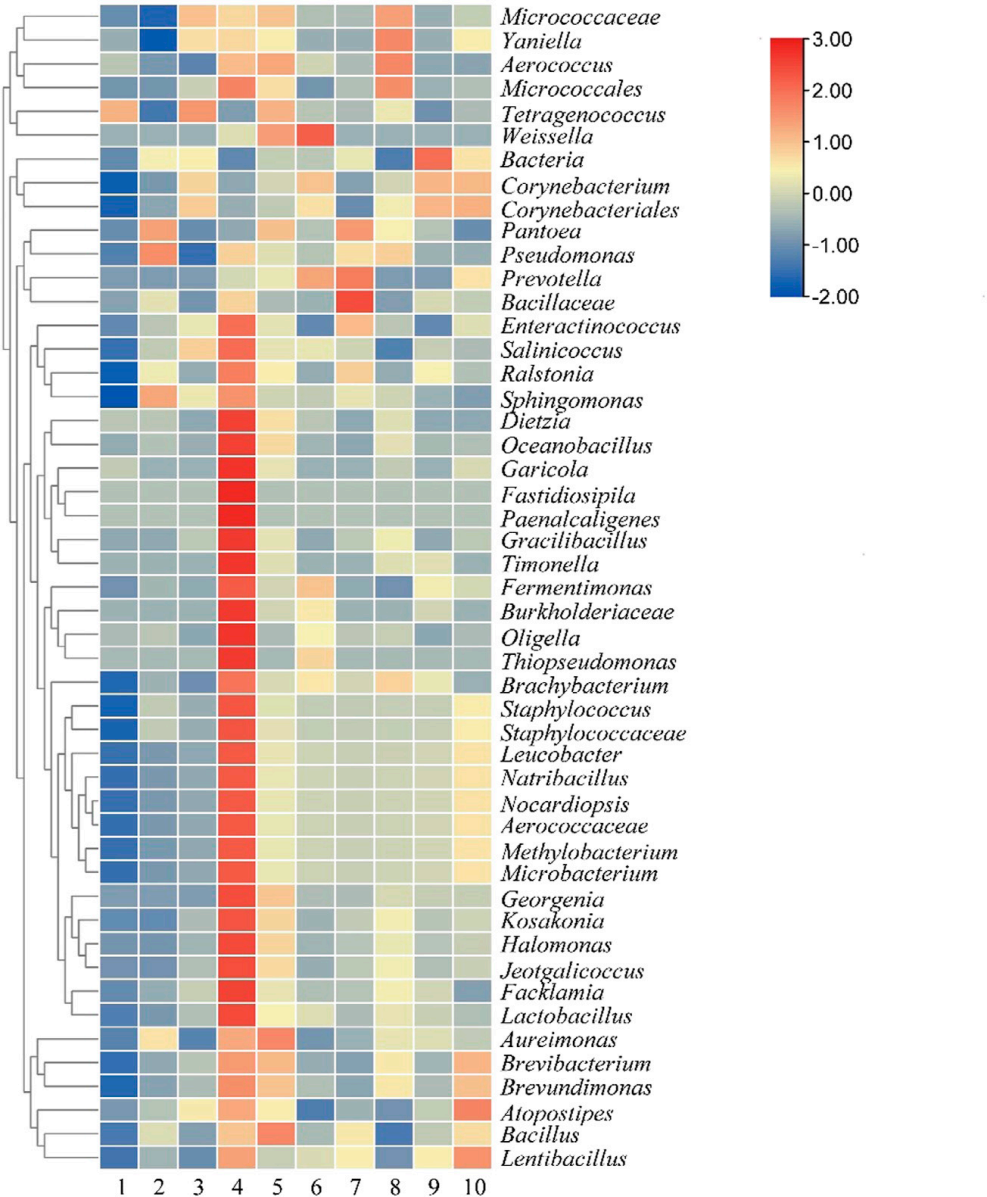
Microbial community of cigar tobacco leaves at genus level. Note: 1 to 5 were respectively added water and fermented for 0 d, 2 d, 4 d, 6 d, 8 d, 6-10 were followed by medium fermentation for 0 d, 2 d, 4 d, 6 d, 8 d.

At the genus level, a total of 201 genera were detected. It can be seen from Figure 7 that the number of most bacteria and genera reached a peak on the sixth day of fermentation in the control group, and then decreased, which was consistent with the change trend of the main aroma components detected above. The dominant genera at the end of the fermentation were *Bacillus*, *Aureimonas*, *Micrococcaceae,* and *Brevibacterium*. In the test group, no obvious growth trend of bacteria was found. As the fermentation progressed, the main dominant bacteria were *Micrococcus*, *Bacillus*, *Prevotella,* and *Aerococcaceae*. After 8 days of fermentation, The dominant bacterial genera in tobacco leaves were *Bacillus*, *Brevibacterium*, *Lentibacillus,* and *Brevundimonas*. The common bacterial genera in the two groups of tobacco leaves after fermentation were *Leucobacter*, *Microbacterium*, *Methylobacterium*, *Staphylococcus*, *Bacillus,* and *Brevibacterium*.

### 3.7 Functional gene prediction analysis

**FIGURE 8:**
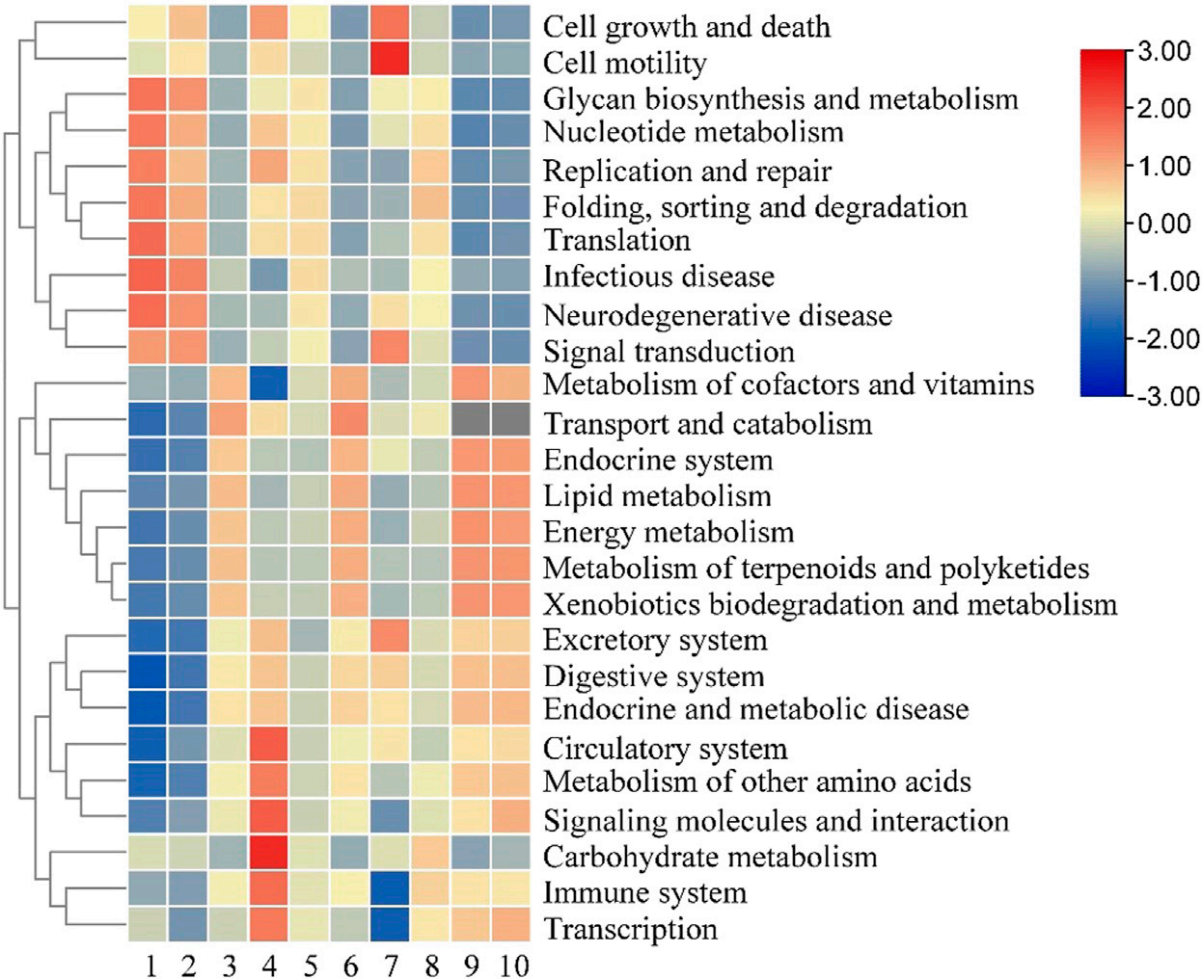
Analysis of bacterial genes in cigar tobacco leaves. Note: 1 to 5 were respectively added water and fermented for 0 d, 2 d, 4 d, 6 d, 8 d, 6-10 were followed by medium fermentation for 0 d, 2 d, 4 d, 6 d, 8 d.

Based on the analysis of the bacterial community distribution of the two groups of fermented tobacco leaves in different periods, the functional genes were further analyzed. All bacterial gene sequences were annotated into 26 functional categories, and the abundance of each functional category was analyzed. It can be seen from Figure 8 that in the early stage of fermentation, the relative abundance of genes related to cell growth, transcription, and translation in the control group was relatively high. The expression levels of related genes reached a peak in the middle stage of fermentation, and then began to decline; in the experimental group, the expression levels of growth-related genes were low in the early stage of fermentation, showing a trend of first increase and then decrease, and energy metabolism, terpenoid polyketone substances. The expression levels of metabolism and other amino acid metabolism-related genes first decreased and then increased, and remained at a high level in the late fermentation stage. In the later stage of fermentation, the relative abundance of genes related to vitamin and amino acid metabolism in the experimental group was higher than that in the control group, and the expression levels of genes related to cell growth activity were significantly lower than those in the control group, which may be the total amount of main amino acids in the experimental group after fermentation. The main reason is lower than the control group.

## 4 Discussion

During the fermentation process of flue-cured tobacco leaves, the method of adding exogenous substances to increase the aroma of tobacco leaves, improve the taste of tobacco leaves, and improve the quality of tobacco leaves is widely used. In this study, adding a medium for fermentation to cigar leaves, allowing the medium to participate in the alcoholization process of cigar leaves, and improving the microbial activity in tobacco leaves can make up for some of the defects in cigar leaves after agricultural fermentation and improve the quality of tobacco leaves (Zhang et al., 2020) Carbohydrates, proteases, amino acids, etc., contained in the medium are involved in the alcoholization of tobacco leaves, and are utilized by microorganisms in tobacco leaves to produce some beneficial metabolites (Wang et al., 2015; Mo et al., 2022).

During the fermentation process of tobacco leaves, the overall fluctuation of protein content in the control group and the test group was small. Therefore, when selecting the fermentation medium, the protein-containing medium should be reduced to avoid being degraded by the microorganisms of the tobacco leaves after addition, thereby affecting the taste of the cigar. The medium with protein-reducing function can be selected to reduce the protein content after fermentation, which is beneficial to improve the smoking taste of tobacco leaves (Yang et al., 2017). In the early stage of fermentation, the content of amino acids in the experimental group was higher than that in the control group with water, probably because the medium itself contained some amino acids. The amino acid reduction in the medium group was higher than that in the water-added group at the late stage of fermentation, probably because the microbial metabolic activity was enhanced after adding the medium, which was beneficial to enhance the microbial activity in tobacco leaves and accelerate the alcoholization of tobacco leaves. Studies have shown that the addition of feed liquid helps to increase the content of flavor amino acids such as glycine, alanine, and proline in tobacco leaves, which may improve the flavor of cigar leaves (Dong et al., 2000). In the two groups of tobacco leaves, the content of petroleum ether extracts increased first and then decreased, which may be due to the high microbial activity in the tobacco leaves at the early stage of fermentation, and some macromolecular substances were degraded and converted into petroleum ether extracts such as fatty acids and esters. The peak time of petroleum ether extract content was different, indicating that the longer the fermentation time of tobacco leaves, the better, and it is necessary to explore different optimal fermentation times when using different media for fermentation. Studies have shown that the content of myristic acid is positively correlated with the aroma and taste of tobacco leaves, and the content of linolenic acid and linoleic acid is negatively correlated with the aroma and taste of tobacco leaves. It is lower, indicating that fermentation is beneficial to increase the aroma and taste of tobacco leaves and reduce the irritation of tobacco leaves (Wang et al., 2015).

The bacterial diversity on the surface of tobacco leaves during fermentation was analyzed by 16S rDNA high-throughput sequencing technology, and it was found that there were significant changes in the community structure and dominant flora of tobacco leaves in different periods. In the two groups of experiments, the overall species richness and community diversity of the control group were slightly higher than those of the control group, probably because the medium itself contained certain microorganisms that participated in the alcoholization during the fermentation process. In the early stage of fermentation, the number of microorganisms in the control group was less, and the number of microorganisms gradually increased as the fermentation progressed, reaching a peak on the 6th day, and then began to decline. In the test group, the initial number of microorganisms was relatively large, and the number of microorganisms reached the peak on the second day of fermentation, and then began to decline, which indicated that the addition of medium was beneficial to the growth of microorganisms, which was helpful to accelerate the alcoholization of tobacco leaves and shorten the fermentation time. In the two groups of samples, there are differences in the dominant flora. The number of microorganisms in the control group was small at the initial stage, and gradually increased as the fermentation progressed. The dominant flora in the later stage of fermentation were mainly Bacillus and Aeromonas. This is consistent with Zhang Ge et al. The cigar cladding predominant bacteria genus had the same results. In the test group, Wessilla was the main species in the early stage of fermentation, Bacillus and Prevotella in the middle stage of fermentation, and Bacillus slow-growing in the late stage of fermentation. This is consistent with the research results of Du et al. (2016) and Ye et al. (2021). The main bacteria after fermentation of tobacco leaves are Bacillus, mainly because the spore-producing bacteria have better stress resistance (Ruan et al., 2005). From the perspective of gene expression levels during the fermentation process, the relative abundances of functional genes related to transport catabolism, energy cycle, lipid metabolism, biodegradation and metabolism in the test group were higher than those in the control group at the early stage of fermentation, and were similar to those in the latter stage of fermentation. The relative abundance of genes related to terpenoid and polyketone metabolism and other amino acid metabolism remained at a high level. It may be that after adding the medium, the nutrients available to tobacco leaf microorganisms increased, and the cell metabolism was enhanced (Tang et al., 2008; Li et al., 2010).

At present, there are few studies on the use of medium for fermentation in cigar tobacco leaves, and the changes of microbial community and the mechanism of action during cigar fermentation are still unclear. Later, the use of medium fermentation to improve the quality of cigar leaves laid the foundation.

## 5 Conclusion

The results of the study showed that by adding medium to ferment cigar leaves, the overall changes in protein and amino acid contents were not significant. The content of petroleum ether extract in the addition test group was significantly lower than that in the control group. Among the fatty acid composition of tobacco leaves, the saturated fatty acid content in the addition test group increased. The amplitude was smaller than that of the control group, and the decrease of the relative content of unsaturated fatty acids was smaller than that of the control group. The species richness and community diversity in the feeding experimental group were slightly higher than those in the control group. The changes of chemical components in cigar leaves were related to the microbial diversity on the surface of tobacco leaves. The addition of media directly affected the dominant flora in tobacco leaves and improved the microbial activity of tobacco leaves. The changes of microbial community and the mechanism of action during the fermentation of cigar tobacco leaves by adding medium are not yet clear, and further research is needed.

## Data Availability

The original contributions presented in the study are included in the article/supplementary material, further inquiries can be directed to the corresponding author.
